# rSjP40 Inhibited the Activity of Collagen Type I Promoter *via* Ets-1 in HSCs

**DOI:** 10.3389/fcell.2021.765616

**Published:** 2021-11-08

**Authors:** Jing Li, Jiali Zhang, Bei Zhang, Liuting Chen, Guo Chen, Dandan Zhu, Jinling Chen, Lian Duan, Yinong Duan

**Affiliations:** ^1^ Department of Pathogen Biology, School of Medicine, Nantong University, Nantong, China; ^2^ Cancer Research Center Nantong, Nantong Tumor Hospital & Tumor Hospital Affiliated to Nantong University, Nantong, China; ^3^ Department of Laboratory, Xishan People’s Hospital of Wuxi City, Wuxi, China; ^4^ Department of Medical Informatics, School of Medicine, Nantong University, Nantong, China

**Keywords:** schistosoma japonicum protein P40, hepatic stellate cells, collagen type I alpha I, liver fibrosis, ETS-1

## Abstract

Liver fibrosis is a severe disease characterized by excessive deposition of extracellular matrix (ECM) components in the liver. Activated hepatic stellate cells (HSCs) are a major source of ECM and a key regulator of liver fibrosis. Collagen type I alpha I (COL1A1) is one of the main components of ECM and is a major component in fibrotic tissues. Previously, we demonstrated that soluble egg antigen from *Schistosoma japonicum* could inhibit the expression of COL1A1 in activated HSCs. In addition, studies have found that Ets proto-oncogene 1 (Ets-1) suppresses the production of ECM by down-regulating matrix related genes such as *COL1A1* induced by transforming growth factor β, and ultimately inhibits liver fibrosis. In this study, the major aim was to investigate the effect and mechanism of Ets-1 on inhibiting *COL1A1* gene promoter activity in HSCs by recombinant *Schistosoma japonicum* protein P40 (rSjP40). We observed the rSjP40 inhibited the expression of *COL1A1* by inhibiting the activity of the *COL1A1* promoter, and the core region of rSjP40 acting on *COL1A1* promoter was located at -1,722/-1,592. In addition, we also demonstrated that rSjP40 could promote the expression of Ets-1, and Ets-1 has a negative regulation effect on the *COL1A1* promoter in human LX-2 cells. These data suggest that rSjP40 might inhibit the activity of *COL1A1* promoter and inhibit the activation of HSCs by increasing the expression of transcription factor Ets-1, which will provide a new experimental basis for the prevention and treatment of liver fibrosis.

## Introduction

Liver fibrosis is a common pathological change of chronic liver disease, which usually occurs after chronic liver injury. It is known that the main processes of liver fibrosis are the activation of hepatic stellate cells (HSCs) and subsequently the excessive deposition of extracellular matrix (ECM) components (Udomsinprasert and Jittikoon, 2019). After the inflammation continues to develop in the liver, HSCs are activated from a resting state to an activated myofibroblast phenotype (Yin et al., 2013; Fabregat and Caballero-Diaz, 2018), which then leads to an imbalance of the formation and degradation of ECM proteins (Tang, 2015). Collagen is the main component of extracellular matrix and the dominant component in fibrotic tissue (Yang et al., 2017). The major types of collagen in liver fibrosis include type I, III and IV. The massive deposition of collagen type I alpha I (COL1A1) and collagen III in the interstitium is a sign of advanced hepatic fibrosis (Chatterjee et al., 2005). In the early stage of fibrosis, collagen III is slightly increased, while COL1A1 is highly increased, and it is still the main type of collagen later on (Eckes et al., 2000). Therefore, the expression level of COL1A1 can reflect the progression of liver fibrosis, inhibiting its expression will be one of the effective measures to reduce liver fibrosis.

Schistosomiasis is a common parasitic disease induced by schistosomes. The main pathogenesis of schistosomiasis is the occurrence of egg granuloma in the liver and intestinal wall, which can lead to severe enterohepatic fibrosis (Duan et al., 2014). Soluble egg antigen (SEA) contains a variety of antigen components and *Schistosoma japonicum* protein P40 (SjP40) is one of the main components in the SEA, which belongs to the heat shock protein family of schistosomiasis (Zhu et al., 2018). Previous studies in our lab have shown that SEA from *Schistosoma japonicum* could inhibit the expression of COL1A1 in activated HSCs (Duan et al., 2014). We also confirmed that the recombinant SjP40 protein (rSjP40) could inhibit the activation and proliferation of HSCs (Sun et al., 2015). However, the contribution and the mechanism of SjP40 in inhibiting the expression of COL1A1 in activated HSCs have not been demonstrated.

Ets proto-oncogene 1 (Ets-1), a member of the Ets transcription factor family, has been reported to be involved in fibrosis progression by regulating fibroblast activation and proliferation, and regulating the synthesis and degradation of ECM. Studies have found that up-regulation of Ets-1 expression enhances transforming growth factor *β* (TGF-β)-induced hepatocyte apoptosis and accelerates liver inflammation and fibrosis in nonalcoholic steatohepatitis (NASH) mice (Liu et al., 2019). However, it has been reported that Ets-1 suppresses the production of ECM by down-regulating matrix related genes such as *COL1A1* induced by TGF-β, and ultimately inhibit liver fibrosis (Ozaki et al., 2002; Ozaki et al., 2003; Liu et al., 2016). However, the role of Ets-1 in the inhibition of COL1A1 expression by rSjP40 remains unclear.

In this study, we observed that whether the transcription factor Ets-1 was participated in the inhibition of COL1A1 in human HSC cell lines LX-2 cells treated with rSjP40.

## Materials and Methods

### Reagents

We obtained rSjP40 protein as previously described (Chen et al., 2016b). Mouse mAbs against COL1A1 (Abcam, United Kingdom), rabbit mAbs against GAPDH (Goodhere, China) or Ets-1 (Proteintech, United Kingdom), horseradish peroxidase (HRP)-conjugated anti-mouse IgG (Santa Cruz, United States) and HRP-conjugated anti-rabbit IgG (Biosharp, China) were purchased from the indicated companies. Plasmids containing *COL1A1* promoter sequences constructed as previously described (Chen et al., 2019a) were preserved in our lab.

### Cell Culture and Treatment

LX-2 cell, a human HSC line, were obtained from Nantong Third People’s Hospital and cultured in Dulbecco’s Modified Eagle’s Medium (DMEM, Gibco, United States) supplemented with 10% FBS (Excel, China). Cells were maintained in a humidified incubator containing 5% CO_2_ at 37°C, inoculated into 12 or 24 well culture-plates and then treated with rSjP40.

### Western Blot

Cell lysates were prepared on ice for extracting proteins using RIPA buffer containing protease inhibitor (1 mM) and phosphatase inhibitors (1 mM). An equal amount of each lysate (50 μg) was separated by 10% sodium dodecyl sulfate-polyacrylamide gel electrophoresis. After the proteins were transferred onto polyvinylidene difluoride (PVDF, Millipore, United States) membranes, the membranes were blocked in TBST containing 5% nonfat milk for 1 h and then incubated with the indicated primary antibodies diluted with 5% nonfat milk overnight at 4 C. The primary antibodies used for the western blot were as follows: mouse antibody against COL1A1 (Abcam, United Kingdom) (1:200 dilution), and rabbit antibody against Ets-1 (Proteintech, United Kingdom) (1:500 dilution) or GAPDH (Goodhere, China) (1:1,000 dilution). After being washed for five times with TBST for 10 min, the membranes were incubated with the indicated secondary antibodies (horseradish peroxidase (HRP)-conjugated anti-mouse IgG (Santa Cruz, United States) and HRP-conjugated anti-rabbit IgG (Biosharp, China) (1:5,000 dilution)) for 1 h at room temperature. Then the protein bands were visualized with an ECL reagents (Millipore, United States) using Image Lab software (Bio-Rad Laboratories Inc., Hercules, CA, United States). GAPDH was viewed as an internal control. Finally, Image J (National Institute of Mental Health, United States) software was used to quantify the intensity of the protein bands.

### Dual-Luciferase Reporter Assay

The indicated plasmids of *COL1A1* promoter and the pRL-TK reporter plasmids were cotransfected into LX-2 cells according to the manufacturer’s instructions of FuGENE (Promega, United States). After transfection for 18 h, LX-2 cells were treated with rSjP40 or not. The cells were then harvested after 48 h of stimulation. Dual-luciferase reporter assay was performed and the firefly and renilla luciferase activities were detected.

### Ets-1 Interference Experiment

LX-2 cells were seeded in a twelve-well plate at a density of 5×10^4^ cells per well. When cultured to 70–90% confluency, 4 μL of lipofectamine 2000 reagent (Invitrogen, United States) was diluted with 100 μL of DMEM and incubated at room temperature for 5 min. Next, 2 μL of siRNA of Ets-1 or negative control siRNA were combined with lipofectamine 2000/medium mixture and allowed to complex by incubation for 20 min at room temperature. The mixed solution was added to 1 ml of cell in the twelve -well plate. After transfection for 4–5 h, the culture medium was replaced with the fresh medium and the cells were incubated in the presence or absence of 20 μg/ml rSjP40 for another 48 h.

### Chromatin Immunoprecipitation (ChIP)

ChIP assay was carried out by SimpleChip Kit (Cell Signaling Technology, United States). Immunoprecipitation was performed with anti-Ets-1 antibody at 4°C overnight and normal IgG provided in SimpleChip Kit was used as a negative control. Precipitated DNA was analyzed by PCR using the primers as: 5′-CAA​TGG​AAT​CTT​GGA​TGG-3’ (sense); 5′- TGA​GAA​ACT​CTG​TAG​GGC-3’ (antisense), which were designed based on the Ets-1-binding sites on the *COL1A1* promoter.

### Statistical Analysis

All experiments were analyzed by the Student’s t test. All values included in the figures represent mean ± SD. A *p*-value < 0.05 was considered significant.

## Results

### rSjP40 Down-Regulated the Activity of *COL1A1* Promoter in LX-2 Cells

**FIGURE 1:**
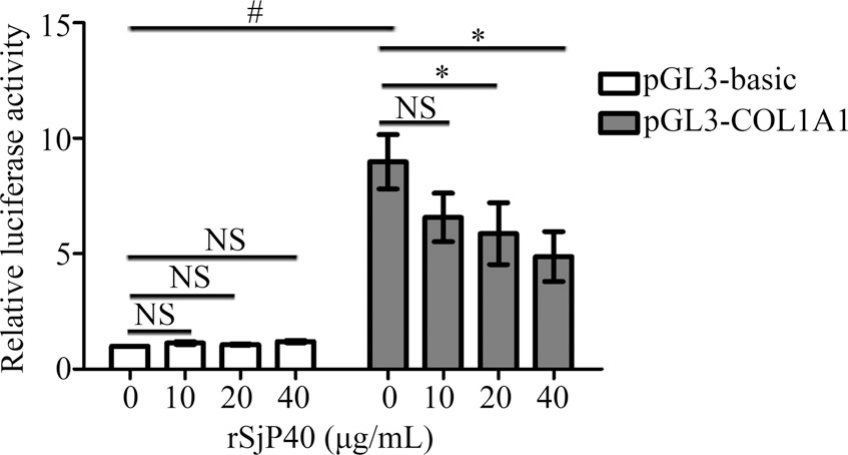
rSjP40 inhibited COL1A1 promoter activity in LX-2 cells. The activity of COL1A1 promoter in LX-2 cells transfected with pGL3-COL1A1 was obviously enhanced than the luciferase activity in LX-2 cells transfected with pGL3-basic. *p* < 0.05, compared to pGL3-basic+ rSjP40-group. rSjP40 could inhibit the activity of COL1A1 promoter in LX-2 cells transfected with pGL3-COL1A1. However, rSjP40 could not affect the luciferase activity in LX-2 cells transfected with pGL3-basic. *p* < 0.05, compared to pGL3-COL1A1+ rSjP40-group. NSP>0.05, compared to pGL3-COL1A1+ rSjP40-group or pGL3-basic + rSjP40- group. NS, no significant.

In our previous studies, we have confirmed that rSjP40 could inhibit COL1A1 expression in LX-2 cells (Sun et al., 2015; Chen et al., 2016b). *COL1A1* promoter is located upstream of transcription initiation and plays a key role in regulating *COL1A1* transcription. In this study, we further investigated the effect of rSjP40 on the transcriptional activity of *COL1A1* expression. We constructed the *COL1A1* promoter plasmid pGL3-COL1A1 (-1,722/+21). The fluorescence reporter plasmid containing *COL1A1* promoter was transfected into LX-2 and detected by dual luciferase reporter assay. Dual luciferase reporter gene assay showed that significantly higher activity was observed in pGL3-COL1A1 than pGL3-basic (*p* < 0.05, Figure 1). In addition, rSjP40 inhibited *COL1A1* promoter activity in a concentration-dependent manner (Figure 1). Hence, we consider that rSjP40 inhibits COL1A1expression in LX-2 cells by inhibiting the activity of *COL1A1* promoter.

### rSjP40 Down-Regulated the Promoter activity of *COL1A1* at the Core Region of -1,722/-1,592

**FIGURE 2:**
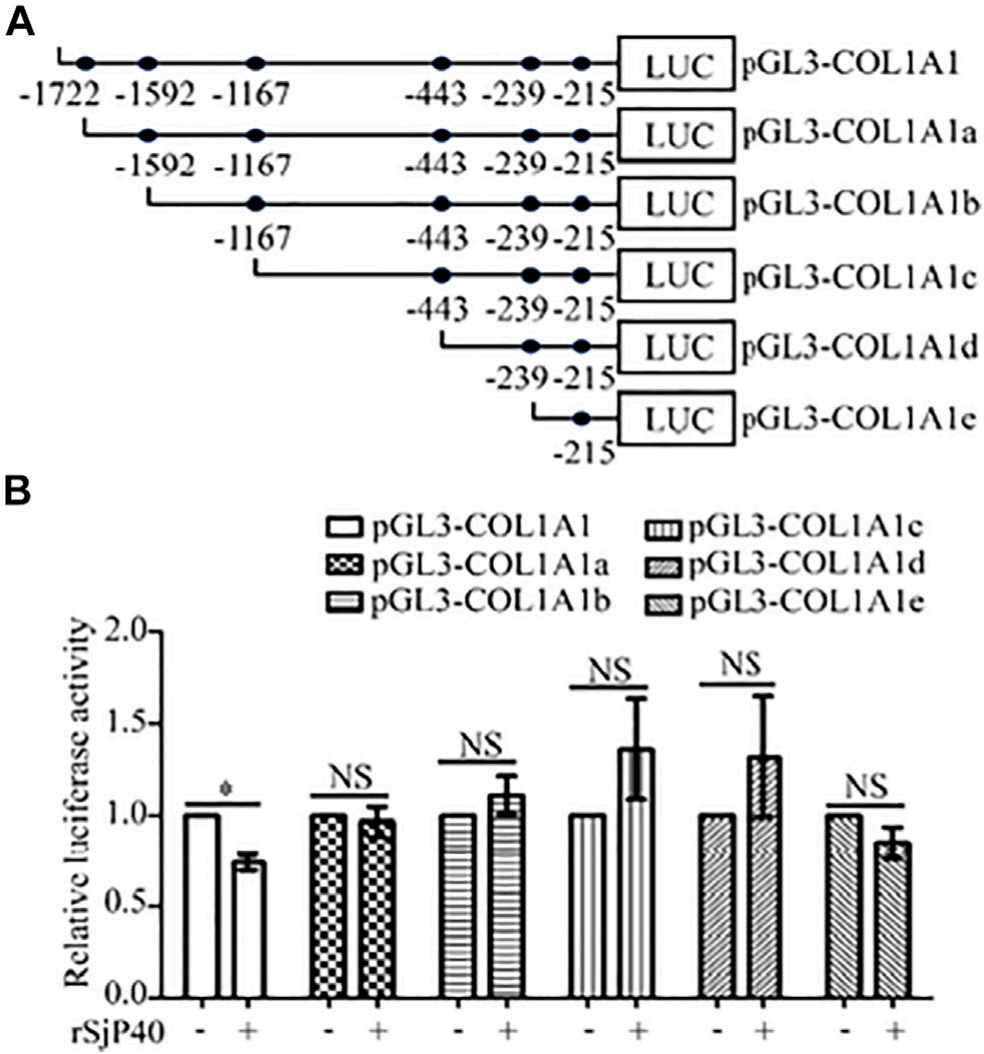
rSjP40 down-regulated the promoter activity of *COL1A1* at the core region of - 1,722/-1,592. **(A)** Diagram of the construction of *COL1A1* promoter truncated fragments. **(B)** The activity of *COL1A1* promoter in LX-2 cells transfected with pGL3-COL1A1 was obviously inhibited by rSjP40. *p < 0.05, compared to pGL3-COL1A1+ rSjP40-group. However, rSjP40 could not affect the luciferase activity in LX-2 cells transfected with the empty pGL3-basic vector, pGL3-COL1A1, pGL3-COL1A1a, pGL3-COL1A1b, pGL3-COL1A1c, pGL3-COL1A1d or pGL3-COL1A1e reporter plasimid. NSP >0.05, compared to each control group with no rSjP40 stimulation. NS, no significant.

Further, we attempted to seek the core region at which rSjP40 could inhibit the promoter activity of *COL1A1*. We used bioinformatics to analyze *COL1A1* promoter sequences and predict possible binding sites for common transcription factors. Truncated mutation of transcription factor binding site was carried out and five luciferase reporter gene plasmids were constructed for experimental study (Chen et al., 2019a): pGL3-COL1A1a (-1,592/+21), pGL3-COL1A1b (-1,167/+21), pGL3-COL1A1c (-443/+21), pGL3-COL1A1d (-239/+21), pGL3-COL1A1e (-215/+21) (Figure 2A). Then the plasmids containing the truncated sequences of *COL1A1* promoter (Chen et al., 2019a) were transfected into LX-2 cells, respectively. We found that rSjP40 could only inhibit the promoter activity in cells transfected with pGL3-COL1A1 (Figure 2B). However, rSjP40 could not affect the promoter activities in cells transfected with these truncated plasmids (Figure 2B). Compared the sequences of pGL3-COL1A1 and pGL3-COL1A1a (Chen et al., 2019a), we confirmed that rSjP40 down-regulated the promoter activity of *COL1A1* at the core region of -1,722/-1,592.

### Ets-1 May Bind to *COL1A1* Promoter at the Region of -1,679/-1,673

**FIGURE 3:**
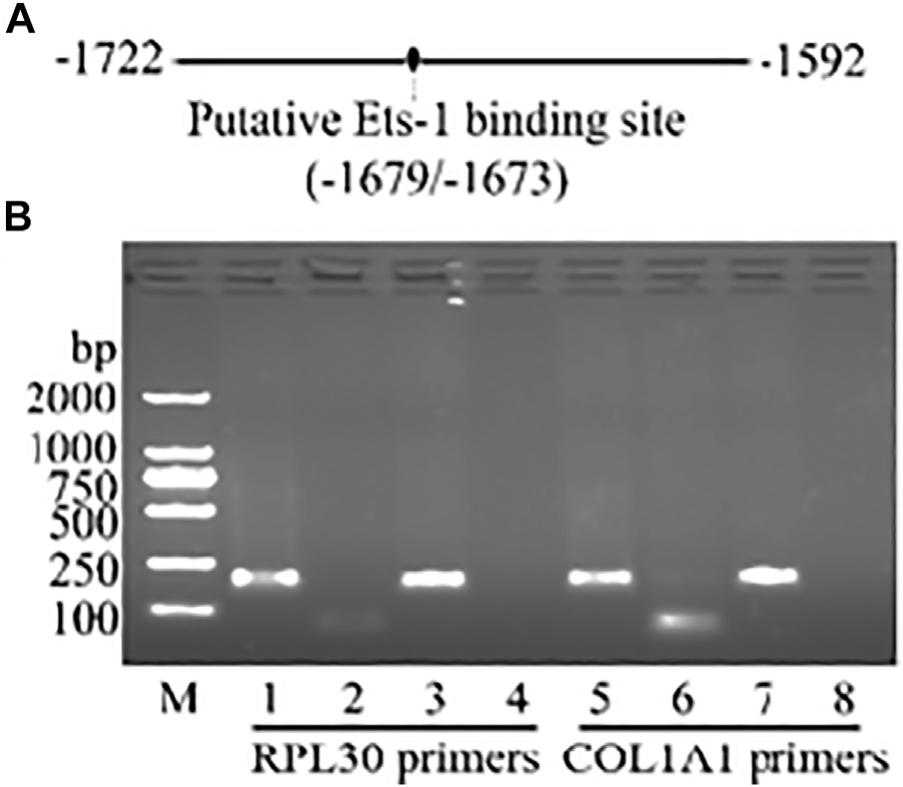
Ets-1 may bind to *COL1A1* promoter at the region of -1,679/-1,673. **(A)** The binding site of transcriptional factor Ets-1 was predicted *via* JASPAR. **(B)** The combination of Ets-1 and collagen type I promoter was confirmed by ChIP method. Lanes 1–4, using RPL30 primers in the kit as the quality control experiment. Lanes 5–8, using COL1A1 promoter primers which were associated with Ets-1 binding site. Lane 1, anti-Histone H3 group. Lane 5, anti-Ets-1 group. Lane 2 and lane 6, IgG group. Lane 3 and lane 7, Input. Lane 4 and lane 8, ddH_2_O as the template of PCR. M, DL2000 DNA marker.

To further explore whether transcription factors are involved in rSjP40 inhibiting *COL1A1* promoter activity, we used JASPAR and PROMO database to predict the possible transcription factor binding sites in the main active region of *COL1A1* promoter -1,722/-1,592. The predicted transcription factor was Ets-1, which was located at -1,679/-1,673 and shared similar loci in both databases (Figure 3A). Therefore, we hypothesized that rSjP40 might inhibit COL1A1 expression in LX-2 cells by regulating the transcription factor Ets-1. To verify this combination, ChIP analysis was performed and the results shown in Figure 3B confirmed that Ets-1 indeed combined to *COL1A1* promoter at the region of -1,679/-1,673. To perform the quality control experiment of ChIP kit, anti-Histone H3 antibody was used as the positive control and the template was then harvested to perform PCR using RPL30 primers provided in the ChIP kit.

### rSjP40 Inhibited the Expression of COL1A1 *via* Ets-1

**FIGURE 4:**
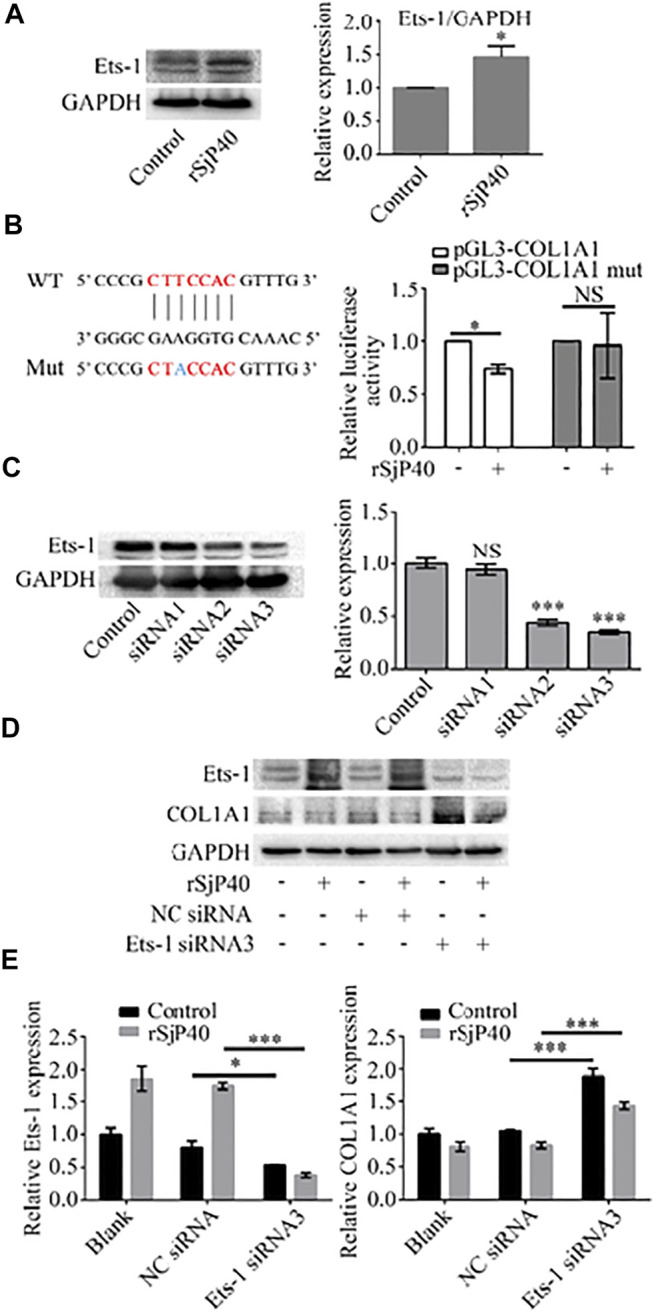
rSjP40 inhibited the expression of COL1A1 *via* Ets-1. **(A)** rSjP40 could enhance the expression of Ets-1 protein in LX-2 cells. ^*^ *p* < 0.05, compared to control group. **(B)** Diagram of SP1-binding site in the *COL1A1* promoter region -1,679/-1,673 and Ets-1 binding site mutant was showed **(left)**. Red represents the ETS-1 binding region and blue represents the mutation site. rSjP40 could inhibit the luciferase activity of *COL1A1* promoter (pGL3-COL1A1), but not inhibit the luciferase activity of the mutated *COL1A1* promoter (pGL3-COL1A1 mut) **(right)**. NSP >0.05, compared to control group with no rSjP40 stimulation. **(C)** siRNA NC or siRNA Ets-1 was transfected into LX-2 cells. The downregulated Ets-1 was confirmed by western blot 72 h post transfection. **(D)** Western blot results showed that a higher level of COL1A1 in siRNA Ets-1 cells than that in the siRNA NC group. **(E)** Quantification of western blot data for Ets-1 and COL1A1 in LX-2 cells. *p < 0.05, *p* < 0.001 compared to siRNA NC group. WT, wild type; Mut, mutant; NS, no significant; NC: Negative control.

Previous studies have shown that Ets-1 is expressed in HSCs and regulates the transcription of ECM genes (Knittel et al., 1999). We further observed the expression of Ets-1 in rSjP40-treated LX-2 cells. The results of Western blot showed that rSjP40 could enhance the expression of Ets-1 in LX-2 cells (Figure 4A). To further confirm that rSjP40 inhibits the *COL1A1* promoter by regulating the transcription factor Ets-1, we mutated the base "T" at the ETS-1 binding site -1,677 on the *COL1A1* promoter into "A" and labeled it as pGL3-COL1A1 mut. The effect of rSjP40 on *COL1A1* promoter was detected by dual luciferin reporter gene. We found that rSjP40 could inhibit the luciferase activity of *COL1A1* promoter (pGL3-COL1A1, Figure 4B). However, when the sequence of -1,679/-1,673 was mutated, rSjP40 could not inhibit the luciferase activity of the mutated *COL1A1* promoter (pGL3-COL1A1 mut, Figure 4B). These results suggested that Ets-1 played a negative regulatory role in the *COL1A1* promoter. Then we further explored whether rSjP40 affects *COL1A1* expression by regulating Ets-1. Successful knockdown of Ets-1 in LX-2 cells were confirmed by western blot (Figure 4C). And knockdown of Ets-1 reversed rSjP40-induced down-regulation of COL1A1 expression (Figures 4D,E). These results indicated that rSjP40 inhibited *COL1A1* promoter activity and COL1A1 expression in LX-2 cells through ETS-1 dependent mechanism.

## Discussion

Liver fibrosis is a typical response to chronic liver disease and is characterized by large and excessive extracellular matrix in the liver. Currently, liver fibrosis is considered to be an evolutionarily conserved wound healing response to tissue injury, primarily driven by inflammatory and immune-mediated mechanisms (Pellicoro et al., 2014). Liver fibrosis is a dynamic and bidirectional process in which the interaction of multiple molecules, pathways and systems determines the self-limiting and dynamic equilibrium of fibrosis (Pellicoro et al., 2014; Sun and Kisseleva, 2015). HSCs are the main effector cells in the process of liver fibrosis. After chronic liver injury, they are transformed into myofibroblast-like cells, which unbalance the formation and degradation of extracellular matrix proteins and release pro-inflammatory and pro-fibrotic factors (Tang, 2015). Activated HSCs are the main source of collagen products, which increase the expression of integrin α5β1, and increases collagen synthesis through interactions between α5β1 and the extracellular matrix (Senoo et al., 2010). COL1A1 plays a dominant role in fibrotic scarring and protects liver cells from various harmful stimuli in the early stages of liver injury (Bourbonnais et al., 2012). However, when sustained damage leads to altered tissue function, excessive scarring can be caused and fibrosis can develop in an adverse direction.

Schistosomiasis is mainly caused by *Schistosoma japonicum* and *Schistosoma mansoni*, which are inflammatory diseases that cause fibrosis and portal hypertension (Chen et al., 2019b). These pathological changes are caused by the secretion of SEA by cercaria in the deposited eggs, which destroys the normal tissues of the host through chronic granulomatous inflammation mediated by T lymphocytes (Pearce and MacDonald, 2002; Ren et al., 2016). However, in addition to causing granuloma through immune mechanism, SEA also has a reverse regulation effect on liver fibrosis. Previous studies in our laboratory confirmed that SEA can induce the senescence and apoptosis of HSCs and inhibit the activation of LX-2 cells to inhibit liver fibrosis (Duan et al., 2014; Chen et al., 2016a). The main component of SEA is SjP40, which is the homologous protein of SmP40, the main egg component of *Schistosoma mansoni*, and they have high homology in amino acid sequence. SjP40 is often deposited in tissues in clusters to form larger granulomas. It has been reported that SjP40 and its antibody can be detected from the host as early as 21 days after the infection of *Schistosoma japonicum*, and is considered as a potential candidate antigen for the early diagnosis of schistosomiasis (Zhou et al., 2010). Then whether SjP40 has an effect on collagen prompted us to further explore the role of SjP40 and prepare recombinant *Schistosoma japonicum* egg protein rSjP40. Our results indicate that rSjP40 can inhibit the expression of COL1A1 in LX-2 cells, which is consistent with our previous results, demonstrating that rSjP40 can inhibit liver fibrosis by inhibiting the synthesis of COL1A1 in HSCs (Chen et al., 2016b). In this study, we focused on exploring the correlation between rSjP40 and the *COL1A1* promoter. The results of this study proved that rSjP40 can inhibit the activity of *COL1A1* promoter. At the same time, it was confirmed for the first time that the core region of rSjP40 acting on *COL1A1* promoter was located at -1,722/-1,592.

As one of the most widely studied transcription factors in the Ets family, Ets-1 is involved in cell proliferation and apoptosis, angiogenesis, and tumor progression (Raouf and Seth, 2000). Studies have shown that Ets-1 is a known effector of mitogen-activated protein kinase (MAPK) pathway and a downstream target of extracellular regulated protein kinase (ERK), which effectively regulates the expression of genes related to endothelial cell growth and migration (Ito et al., 2004). Ets-1 has been shown to be expressed in HSCs and can regulate the transcription of ECM genes (Knittel et al., 1999). However, the role of Ets-1 in promoting or inhibiting liver fibrosis remains controversial. M Mizui et al. (Mizui et al., 2006) showed that overexpression of Ets-1 could inhibit the production of COL1A1 in mesangial cells induced by TGF-β, so Ets-1 is considered as an effective inhibitor of collagen synthesis. In contrast, Dechen Liu et al. (Liu et al., 2019) showed that the knockdown of Ets-1 reduced hepatocyte apoptosis and slowed down the progression of non-alcoholic steatohepatitis, protecting the liver from injury, inflammation and fibrosis. In our study, we found that rSjP40 could promote the expression of Ets-1 in human LX-2 cells. Studies have shown that IL-18 down-regulates collagen expression by activating Ets-1 through ERK pathway and directly inhibiting the activity of collagen promoter in human skin fibroblasts (Kim et al., 2010). Therefore, we hypothesized whether rSjP40 affects *COL1A1* promoter activity by regulating Ets-1. CHIP experiments showed that Ets-1 could directly bind to the *COL1A1* promoter at the site-1,679/-1,673. Then we mutated the base "T" at -1,677 of the Ets-1 binding site on the *COL1A1* promoter into "A", and the activity of the *COL1A1* promoter significantly increased, indicating that Ets-1 has a negative regulation effect on the *COL1A1* promoter in human LX-2 cells. In addition, rSjP40 could not inhibit the activity of the mutated *COL1A1* promoter at the Ets-1 binding site, further confirming the role of rSjP40 by affecting Ets-1. Finally, knockdown of Ets-1 in LX-2 cells reversed rSjP40-induced down-regulation of COL1A1 expression.

In conclusion, this study indicates that rSjP40 inhibits the activity of *COL1A1* promoter by increasing the expression of transcription factor Ets-1, down-regulating the expression of COL1A1, thereby inhibiting the activation of HSCs and inhibiting liver fibrosis. The results of this study will provide a new experimental basis for the prevention and treatment of liver fibrosis.

## Data Availability

The original contributions presented in the study are included in the article/Supplementary Material, further inquiries can be directed to the corresponding authors.
